# Protective effect of nicorandil on myocardial injury following percutaneous coronary intervention in older patients with stable coronary artery disease: Secondary analysis of a randomized, controlled trial (RINC)

**DOI:** 10.1371/journal.pone.0194623

**Published:** 2018-04-16

**Authors:** Norifumi Kawakita, Kentaro Ejiri, Toru Miyoshi, Kunihisa Kohno, Makoto Nakahama, Masayuki Doi, Mitsuru Munemasa, Masaaki Murakami, Kazufumi Nakamura, Hiroshi Ito, on behalf of the RINC investigators

**Affiliations:** 1 Department of Cardiovascular Medicine, Okayama University Graduate School of Medicine, Density and Pharmaceutical Sciences, Okayama, Japan; 2 Department of Cardiology, Fukuyama City Hospital, Hiroshima, Japan; 3 Department of Cardiology, Kagawa Prefectural Central Hospital, Kagawa, Japan; 4 Department of Cardiology, Okayama Medical Center, Okayama, Japan; 5 Department of Cardiology, Okayama Heart Clinic, Okayama, Japan; Kurume University School of Medicine, JAPAN

## Abstract

**Background:**

Our previous study examined an effect of remote ischemic preconditioning (RIPC) or intravenous nicorandil on reduction of periprocedural myocardial injury (pMI) following percutaneous coronary intervention (PCI) in patients with stable coronary artery disease (CAD). We further investigated the effect of RIPC or nicorandil on pMI in older patients.

**Methods:**

Patients with stable CAD who planned to undergo PCI were assigned to a 1:1:1 ratio to control, intravenous nicorandil, or upper-limb RIPC groups. This substudy analyzed patients aged >65 years (n = 282) from the principal cohort. The primary outcome was the incidence of pMI following PCI. We defined pMI as an elevated level of high-sensitive cardiac troponin T or creatine kinase myocardial band 12 or 24 hours after PCI.

**Results:**

We found that pMI following PCI was significantly reduced in the nicorandil group compared with the control group (37.2% vs. 53.7%, multiplicity-adjusted *p* = 0.046), but not in the RIPC group compared with the control group (43.0% vs. 53.7%, multiplicity-adjusted *p* = 0.245). The adjusted odds ratios (95% confidence interval) for pMI in the RIPC and nicorandil groups versus the control group were 0.63 (0.34 to 1.16) and 0.51 (0.27 to 0.96), respectively.

**Conclusion:**

Intravenous nicorandil significantly reduces pMI following PCI in a subgroup of older patients with stable CAD. Phase 3 trials are required to validate our results.

**Trial registration:**

UMIN Clinical Trials Registry UMIN000005607.

## Introduction

Periprocedural myocardial injury (pMI) is a complication of elective percutaneous coronary intervention (PCI) in patients with stable coronary artery disease (CAD) and affects long-term prognosis [1,2]. The potential contributing mechanisms for pMI following elective PCI in patients with stable angina include downstream embolization of atheromatous material and coronary side-branch occlusion [3,4]. However, pMI is associated with various factors, including procedure-related, lesion-related, and patient-related factors [5]. Patient-related factors are involved in multivessel disease, diabetes mellitus, chronic kidney disease and older age, which increase the risk of postprocedural creatine kinase myocardial band (CK-MB) release by 1.3- to 1.8-fold. [6,7,8,9,10]

Several approaches, including remote ischemic preconditioning (RIPC) and nicorandil, have been evaluated to increase cardiac tolerance to ischemic injury following PCI [11,12,13,14,15,16,17,18,19]. RIPC is defined as when transient nonfatal ischemia and reperfusion applied to one organ or tissue protects another organ or tissue from a subsequent episode of fatal ischemia and reperfusion [20]. Nicorandil activates adenosine triphosphate-sensitive potassium channels in mitochondria, and this plays a role in imitating ischemic preconditioning [21,22,23].

We recently reported a multicenter, randomized trial that examined whether pre-procedural RIPC or intravenous nicorandil reduces pMI in patients who undergo elective PCI for stable CAD. This study showed that RIPC or intravenous nicorandil moderately reduced biomarker release and pMI in patients with stable CAD, but these results were not significant [24]. Therefore, an exploratory analysis to identify a population in whom RIPC or intravenous nicorandil is effective was planned. We hypothesized that age influences the effect of RIPC or intravenous nicorandil on pMI. This hypothesis was based on the finding that age was a predictor of pMI [5] and related to short-term and long-term critical events, such as death, in patients who had PCI for acute coronary syndrome [25,26]

This study aimed to investigate the efficacy of RIPC or intravenous nicorandil on pMI in older patients with stable CAD undergoing elective PCI. This was a substudy of our previous multicenter, randomized study.

## Materials and methods

The protocol for this trial, supporting CONSORT checklist, and full data set are available as supporting information (see S1 CONSORT Checklist, S1 Protocol and S1 Data Set).

### Ethics statement

The study was approved by the ethics committees of all hospitals. All participants provided written informed consent before enrolling. This study was conducted according to the principles expressed in the Declaration of Helsinki. The study is registered at the UMIN Clinical Trials Registry (UMIN000005607) at https://upload.umin.ac.jp/cgi-open-bin/ctr_e/ctr_view.cgi?recptno=R000006626. A list of investigators is provided in the Acknowledgments.

### Study design

**Fig 1:**
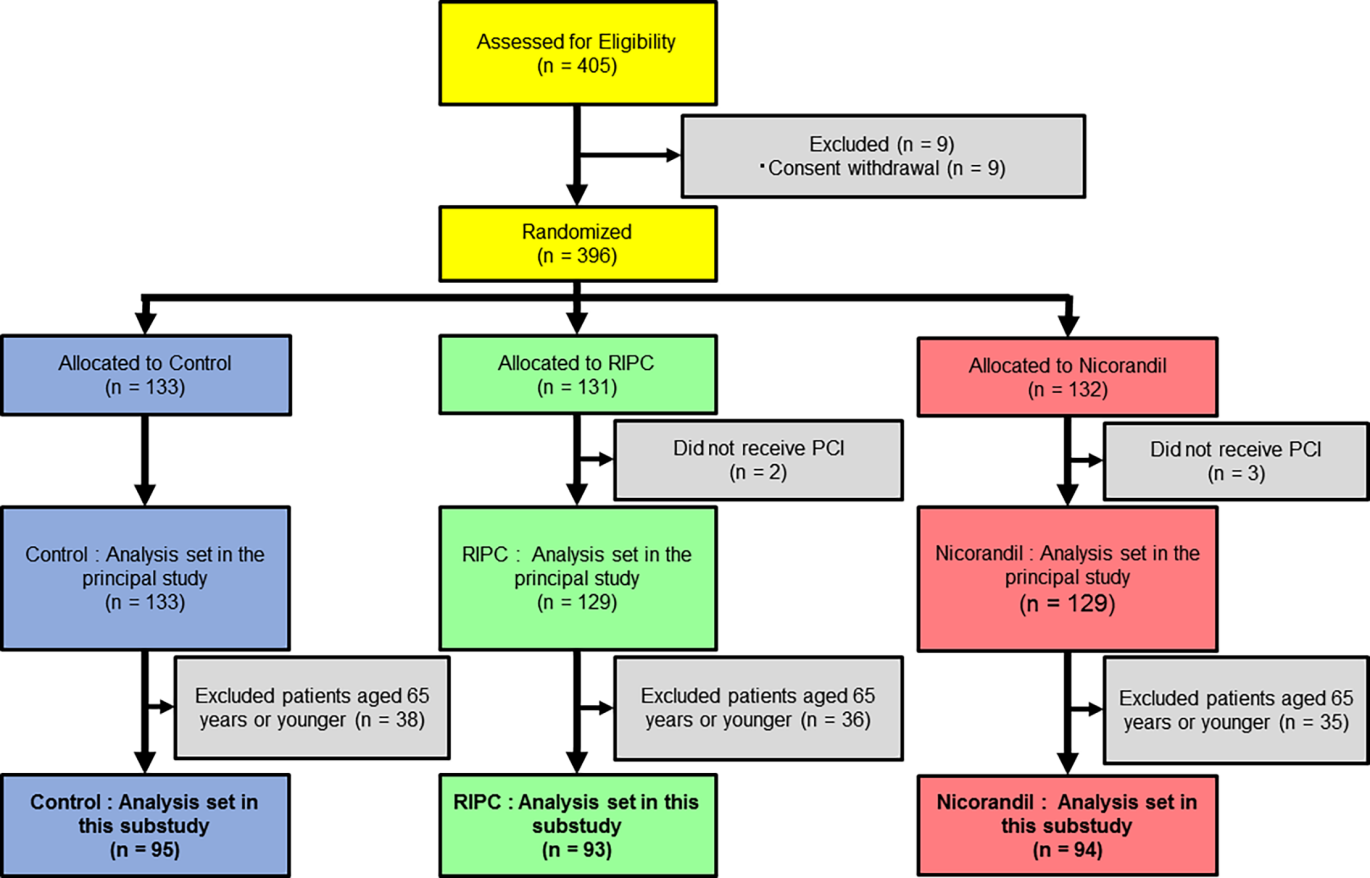
Flow diagram of the study. PCI, percutaneous coronary intervention; RIPC, remote ischemic preconditioning.

The principal study (Cardiac Preconditioning Effect of Remote Ischemia and Nicorandil in Patients Undergoing Elective Percutaneous Coronary Intervention: RINC) was a prospective, open-label, multicenter, randomized, controlled trial, which was conducted between February 2011 and January 2013 [24]. This was a post-hoc analysis of the RINC study. Fig 1 shows a flow diagram of the study. Eligible patients were adults (>20 years old) who were diagnosed with stable CAD, including silent myocardial ischemia and stable angina, and planned to have elective PCI. All of the patients underwent coronary angiography before enrollment in this study. Indication of PCI was evaluated according to the guideline for elective percutaneous coronary intervention in patients with stable coronary artery disease from the Japanese Society of Cardiology [27]. Patients were excluded for the following reasons: they had acute coronary syndrome, there was contraindication of intravenous nicorandil administration, they planned elective PCI for chronic total occlusion lesion or PCI with a rotablator, use of sulfonylurea, an arterio-venous shunt was present in the arms, and prognosis was regarded as less than 12 months. In the principal study, we enrolled 405 patients at 18 coronary intervention centers in Japan. Among them, 396 patients underwent randomization. Using post-hoc analysis, we aimed to identify any myocardial protective effect of RIPC during elective PCI in older patients. Therefore, patients aged 65 years or younger were excluded. Finally, 282 patients were included in this post-hoc analysis (control: n = 95, nicorandil: n = 94, RIPC: n = 93).

The intervention protocol and PCI procedure have been described previously [24]. In patients who were assigned to the nicorandil group, 4 mg of nicorandil was intravenously administered for 5 minutes for at least 1 hour before PCI, followed by continuous infusion of nicorandil (6 mg/h) for at least 8 hours. In patients who were assigned to the RIPC group, 5-minute inflation of a blood pressure cuff to 200 mmHg around the upper arm was performed. This was followed by 5-minute deflation of the cuff to 0 mmHg. This process was performed three times at least 1 hour before PCI. This procedure was automatically performed by a newly developed automated continuous blood pressure device (FB-270; Fukuda Denshi, Tokyo, Japan) [28]. Stopping the infusion was dependent on the practice of each hospital. Patients in the control group did not receive any additional pretreatment before PCI. PCI was performed in a conventional manner.

The primary outcome in the current study was the incidence of pMI following PCI. The definition of pMI was as follows: an elevation in high-sensitive cardiac troponin T (cTnT) levels >0.07 ng/ml (5×99th percentile upper reference limit); or CK-MB levels >10 ng/ml and CK-MB/creatinine kinase levels >5%, at 12 or 24 hours after PCI. If patients were discharged before regular assessment, these cardiac biomarkers were assessed at discharge.

The secondary outcomes in the current study were ischemic events during PCI, including chest pain during PCI, ST segment change on an electrocardiogram (>0.1 mV) during PCI, ventricular arrhythmia required for cardioversion during PCI, and final thrombolysis in myocardial infarction (TIMI) grade. We also studied adverse clinical events at 8 months after PCI as a secondary outcome. Adverse clinical events included cardiovascular or non-cardiovascular death, admission for acute coronary syndrome, any revascularization, and admission for heart failure.

### Statistical analysis

Continuous variables are presented as mean±standard deviation (SD) or as median with the interquartile range. Categorical variables are presented as frequencies and proportions (%). Continuous variables were compared using analysis of variance or the Kruskal–Wallis test for non-normally distributed parameters. Categorical variables were compared using Pearson’s chi-square test.

Fisher’s exact test was applied to compare proportions of pMI following PCI between the nicorandil and control or the RIPC and control groups. The *p* values were adjusted using the bootstrap method (1,000,000 iterations) to account for multiplicity [29]. The odds ratios (ORs) between groups and their 95% exact confidence intervals (CIs) were calculated. Additionally, a logistic regression model was used to calculate ORs between study groups with adjustment for sex, and with or without chronic kidney disease (estimated glomerular filtration rate [eGFR] at baseline <60 or ≥60 ml/min/1.73 m^2^). The adjusted ORs between groups and their 95% exact CIs were calculated. The *p* values were adjusted using the Dunnett–Hsu method to account for multiplicity. The Hosmer–Lemeshow lack-of-fit test was also applied. The same models of the primary outcome were applied to ischemic events during PCI. For analysis of the primary outcome, we also used a mixed-effect logistic regression model in which the center ID was included as a random-intercept term to assess the robustness of the primary analysis. Sandwich estimators of the covariance matrix of regression coefficients were used for the analysis.

For adverse clinical events at 8 months after PCI, the Kaplan–Meier estimate was used by treatment group and compared using the log-rank test. The *p* values were adjusted using the Dunnett–Hsu method to account for multiplicity. The Cox proportional hazards model was used to estimate hazard ratios (HRs) and their 95% profile likelihood CIs between treatment groups. The statistical test of Lin, Wei, and Ying (1993) for checking the adequacy of proportional hazard assumption was also applied [30].

All analyses were performed with IBM SPSS Statistics 24.0 and SAS system 9.4. Two-tailed values of *p*<0.05 were considered statistically significant.

## Results

### Baseline characteristics

Table 1 shows the baseline characteristics of the patients. In patients aged >65 years, 50.0% of patients were diagnosed with diabetes previously. A total of 59.9% of patients were current or past smokers. Procedural characteristics were not significantly different among the groups. A total of 51.1% of patients had complex coronary lesions, such as AHA-ACC characteristics of types B2 (31.9%) and C (19.1%). Target vessels and AHA-ACC characteristics of types of lesions, and the diameter and length of stents were not significantly different among the groups. Baseline characteristics in patients aged ≤65 years (n = 109) are shown in S1 Table.

**Table 1:** Baseline characteristics of patients aged >65 years.

	Control group (n = 95)	RIPC group (n = 93)	Nicorandil group (n = 94)	p value
Age-yr.	75.4(5.3)	75.5(5.9)	74.3(5.9)	0.33
Male-n (%)	69(72.6)	65(69.9)	69(73.4)	0.85
Body mass index (kg/m^2^)	24.0(3.1)	24.0(3.5)	24.6(3.7)	0.36
Angina symptom-n (%)				
symptomatic	65(68.4)	69(74.2)	71(75.5)	0.51
asymptomatic	30(31.6)	24(25.8)	23(24.5)	
Prior diagnoses-n (%)				
Diabetes Mellitus	47(49.5)	43(46.2)	51(54.3)	0.54
Hypertension	86(90.5)	81(87.1)	79(84.0)	0.41
Dyslipidemia	74(77.9)	78(83.9)	79(84.0)	0.46
CCV event history-n (%)	43(45.3)	46(49.5)	44(46.8)	0.84
Smoking history-n (%)				
Current smoker	4(4.2)	5(5.4)	7(7.4)	0.91
Ex-smoker	52(54.7)	50(53.8)	51(54.3)	
Echocardiographic parameters at randomization				
LVEF (%)	61.9(10.7)	61.1(11.5)	63.5(9.0)	0.31
E/e	13.3(5.2)	13.2(5.2)	13.6(4.8)	0.89
Laboratory data at randomization				
Hemoglobin (g/dl)	12.9(1.8)	13.0(1.7)	12.9(1.8)	0.89
Platelet count (10^4^/μl)	19.1(5.7)	20.1(6.1)	20.3(5.9)	0.32
Total cholesterol (mg/dl)	159.0[135.0–185.3]	156.0[137.8–173.0]	162.5[140.0–183.5]	0.28
Serum creatinine (mg/dl)	0.86[0.72–1.0]	0.86[0.75–1.0]	0.85[0.70–1.1]	0.87
eGFR (ml/min/1.73 cm^2^)	63.2(15.1)	61.4(17.5)	61.3(19.1)	0.67
Hemoglobin A1C (%)	5.9[5.3–6.5]	5.7[5.4–6.6]	5.9[5.4–6.6]	0.81
C-reactive protein (mg/dl)	0.10[0.03–0.31]	0.10[0.04–0.22]	0.13[0.06–0.30]	0.31
Brain natriuretic peptide (pg/ml)	37.0[21.4–80.2]	61.5[18.3–129.8]	44.2[23.6–91.4]	0.38
Myocardial biomarker at randomization				
Cardiac troponin T (ng/ml)	0.013[0.009–0.019]	0.012[0.008–0.022]	0.012[0.009–0.020]	0.86
CK-MB (ng/ml)	3.9[2.5–5.2]	3.7[2.7–4.6]	3.8[2.7–5.0]	0.55
Medications at randomization-no. (%)				
Antiplatelets	95(100)	92(98.9)	94(100)	0.36
β-blockers	31(32.6)	39(41.9)	37(39.4)	0.40
ACEIs/ARBs	64(67.4)	54(58.1)	57(60.6)	0.40
Calcium channel blockers	53(55.8)	51(54.8)	43(45.7)	0.31
Statins	71(74.7)	75(80.6)	74(78.7)	0.61
Procedure characteristics				
Target vessel-no. (%)				
LAD	44(46.3)	41(44.1)	37(39.4)	0.62
LCX	15(15.8)	15(16.1)	25(26.6)	0.10
RCA	31(32.6)	32(34.4)	29(30.9)	0.87
multiple	5(5.3)	5(5.4)	3(3.2)	0.72
AHA-ACC classification-no. (%)				
Type A	14(14.7)	15(16.1)	15(16.0)	0.97
Type B1	35(36.8)	31(33.3)	28(29.8)	
Type B2	25(26.3)	30(32.3)	35(37.2)	
Type C	21(22.1)	17(18.3)	16(17.0)	
Amount of contrast medium (ml)	104.3(37.7)	105.7(50.4)	106.7(45.6)	0.94
Puncture site, n/total n (%)				
Radial artery	55/95(57.9)	51/92(55.4)*	57/94(60.6)	0.88
Brachial artery	13/95(13.7)	12/92(13.0)*	14/94(14.9)	
Femoral artery	27/95(28.4)	29/92(31.5)*	23/94(24.5)	
Catheter size, n/total n (%)				
6 Fr	82/95(86.3)	76/91(83.5)**	87/94(92.6)	0.11
7 Fr	11/95(11.6)	15/91(16.5)**	7/94(7.4)	
8 Fr	2/95(2.1)	0	0	
Details of device				
No. of stents used	1[1–2]	1[1–2]	1[1–2]	0.29
Drug-eluting stent, n/total n (%)	123/133(92.5)	113/120(94.2)	114/123(92.7)	0.85
Stent diameter (mm)	2.75[2.5–3.5]	3[2.5–3.5]	3[2.5–3]	0.20
Stent length (mm)	18[15–24]	20[15–28]	20[15–24]	0.33
Stent inflation time (second)	15[10–20]	15[10–20]	15[10–20]	0.26
Post dilation (%)	74/95(77.9)	70/93(75.3)	63/94(67.0)	0.21
Post dilation time (second)	15[10–30]	20[15–30]	15[10–40]	0.96
Maximum dilatation pressure (atm)	16.4(4.4)	17.4(4.3)	16.6(4.5)	0.29

Data are mean (standard deviation), n (%), or median [interquartile range]. RIPC, remote ischemic preconditioning; CCV, cardio-cerebrovascular; LVEF, left ventricular ejection fraction; E, peak velocity of the early diastolic filling wave; e′, mitral annulus velocity; eGFR, estimated glomerular filtration rate; CK, creatine kinase; ACEI, angiotensin-converting enzyme inhibitor; ARB, angiotensin II receptor blocker; LAD, left anterior descending artery; LCX, left circumflex artery; RCA, right coronary artery; AHA, American Heart Association; ACC, American College of Cardiology; Fr, French; atm, atmospheres.

*One patient’s data were uncollected

**two patients’ data were uncollected.

### Primary outcome

**Fig 2:**
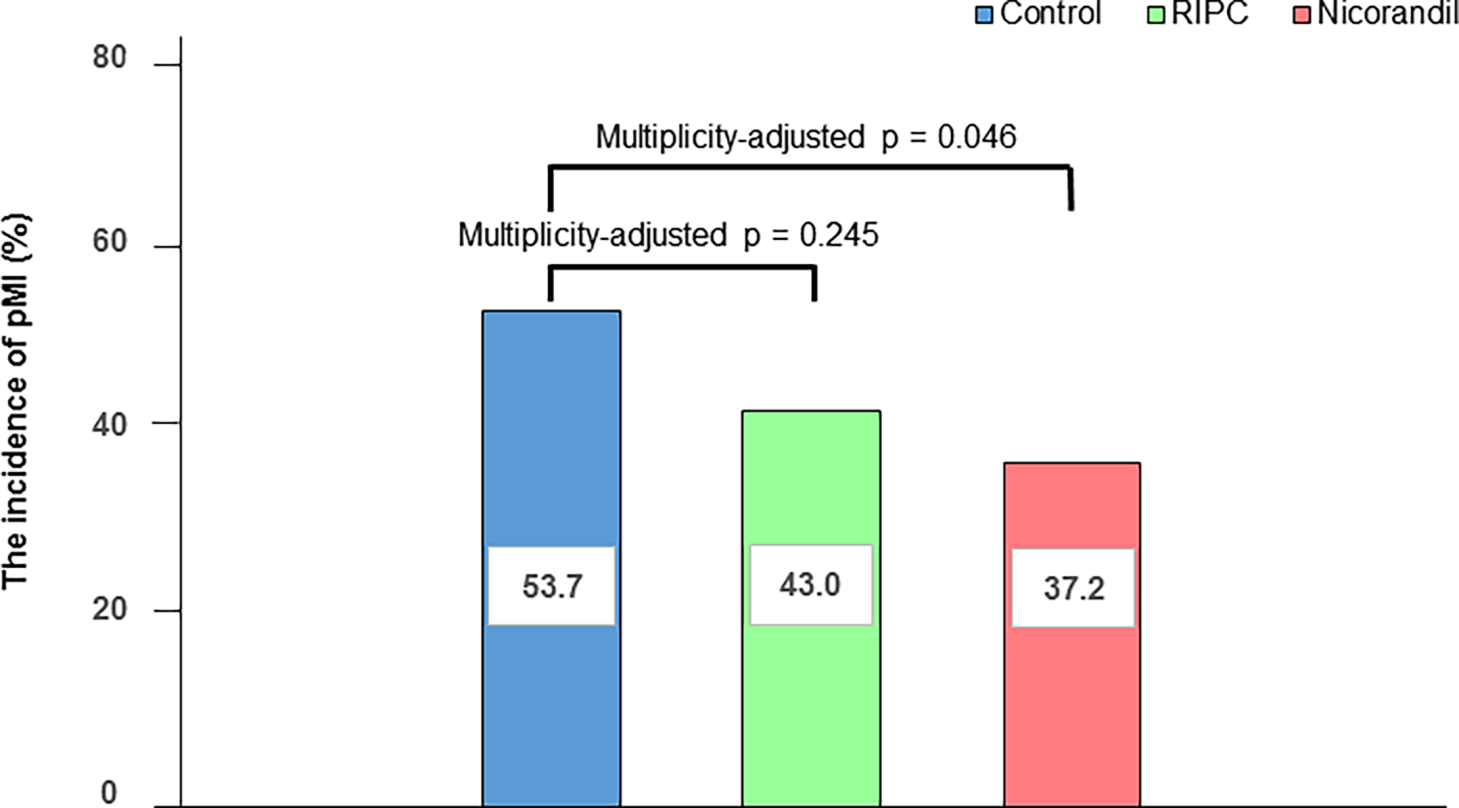
Incidence of periprocedural myocardial injury in patients aged >65 years. pMI, periprocedural myocardial injury; RIPC, remote ischemic preconditioning.

The incidence of pMI following PCI in the nicorandil group (35/94, 37.2%) was significantly lower than that in the control group (51/95, 53.7%) (Fig 2). However, the incidence of pMI was not significantly different between the RIPC and control groups (40/93, 43.0%). In adjusted analysis, the risk reduction remained significant between the control and nicorandil groups (adjusted OR: 0.51; 95% CI: 0.27 to 0.96; multiplicity-adjusted *p* = 0.045) (Table 2). However, the incidence of pMI was not significantly different between the control and RIPC groups (adjusted OR: 0.63; 95% CI: 0.34 to 1.16; multiplicity-adjusted *p* = 0.196). In mixed-effect logistic regression analysis, the incidence of pMI was also significantly different between the control and nicorandil groups (OR: 0.50; 95% CI: 0.31 to 0.82; multiplicity-adjusted *p* = 0.010). However, the incidence of pMI was not significantly different between the control and RIPC groups (OR: 0.65; 95% CI: 0.41 to 1.03; multiplicity-adjusted *p* = 0.119). The *p* values in Hosmer–Lemeshow lack-of-fit tests were non-significant. In patients aged ≤65 years, the incidence of pMI in the nicorandil group (17/35, 48.6%) and in the RIPC group (11/36, 30.6%) was not significantly different compared with that in the control group (14/38, 36.8%) (multiplicity-adjusted *p* = 0.511 and multiplicity-adjusted *p* = 0.745, respectively) (S1 Fig).

**Table 2:** Odds ratios for primary and secondary outcomes in patients aged >65 years.

	Control group	RIPC group	RIPC vs. Control (reference)		Nicorandil group	Nicorandil vs. Control (reference)		Lack-of-fit test
	n = 95	n = 93	OR/HR (95% CI)	Multiplicity-adjusted p-value	n = 94	OR/HR (95% CI)	Multiplicity-adjusted p-value	p value
**Primary end point**								
Perioperative myocardial injury, n (%)								
Unadjusted analysis^a^	51	40	0.65	0.245	35	0.51	0.046	
	(53.7)	(43.0)	(0.35–1.20)		(37.2)	(0.27–0.95)		
Adjusted analysis^b^			0.63	0.196		0.51	0.045	0.861
			(0.34–1.16)			(0.27–0.96)		
**Secondary end points**								
Ischemic events during PCI, n (%)								
Chest pain during PCI	14	16	1.17	0.844	10	0.7	0.558	0.842
	(14.7)	(17.4)*	(0.49–2.82) ^b^		(10.6)	(0.26–1.82) ^b^		
ST segment change on an electrocardiogram	14	19	1.5	0.388	14	1.01	1.000	0.335
	(14.7)	(20.7)*	(0.66–3.49) ^b^		(14.9)	(0.42–2.45) ^b^		
Ventricular arrhythmia needed for cardioversion	0	0*			1			
					(1.1)			
Final TIMI grade, n /total n and (%)								
TIMI 3	95/95	88/90			91/94			
	(100)	(97.8)**			(96.8)			
TIMI 0–2	0	2/90			3/94			
		(2.2)**			(3.2)			
Adverse clinical events for 8 months after PCI, n /total n and (%)								
Total	8/95	7/92	0.87	0.955	11/94	1.38	0.685	RIPC: 0.731, Nicorandil: 0.802
	(10.4)	(8.1)*	(0.31–2.42)^c^		(12.5)	(0.56–3.57)^c^		
All-cause death	0	0			0			
Admission for ACS	0	0			2/94			
					(2.1)			
Revascularization	6/95	3/92	0.49	0.536	7/94	1.16	0.937	RIPC: 0.905, Nicorandil: 0.488
	(8.2)	(3.8)*	(0.10–1.86)^b^		(8.6)	(0.39–3.60)^b^		
Admission for heart failure	2/95	3/92	1.51	0.851	2/94	1.02	1.000	RIPC: 0.771, Nicorandil: 0.617
	(2.4)	(3.3)*	(0.25–11.49)^b^		(2.2)	(0.12–8.48)^b^		
Stroke	0	1/92			0			
		(1.2)*						

RIPC, remote ischemic preconditioning; OR, odds ratio; HR, hazard ratio; CI, confidence interval; TIMI, thrombolysis in myocardial infarction; ACS, acute coronary syndrome.

^a^Fisher’s exact test was used to calculate *p* values. The resulting *p* values were multiplicity-adjusted. Exact 95% CIs for the ORs were also calculated.

^b^A logistic regression model was used to calculate ORs between study groups with adjustment for sex, and with or without chronic kidney disease (baseline eGFR <60 or ≥60 ml/min/1.73 m^2^) in perioperative myocardial injury and ischemic events during PCI. The resulting p values were multiplicity-adjusted. Exact 95% CIs for the ORs were also calculated.

^c^The log-rank test was used to calculate *p* values. The resulting *p* values were multiplicity-adjusted. The Cox proportional hazards model was used to estimate HRs between treatment groups for adverse clinical events for 8 months after PCI. Profile likelihood 95% CIs for the HRs were also calculated.

*One patient’s data were uncollected

**three patients’ data were uncollected.

### Secondary outcomes

During PCI, few ischemic events occurred in each group. The incidence of each event (chest pain during PCI, ST segment change on an electrocardiogram [>0.1 mV] during PCI, ventricular arrhythmia required for cardioversion during PCI, and final TIMI grade) was not significantly different among the control, nicorandil, and RIPC groups (Table 2).

**Fig 3:**
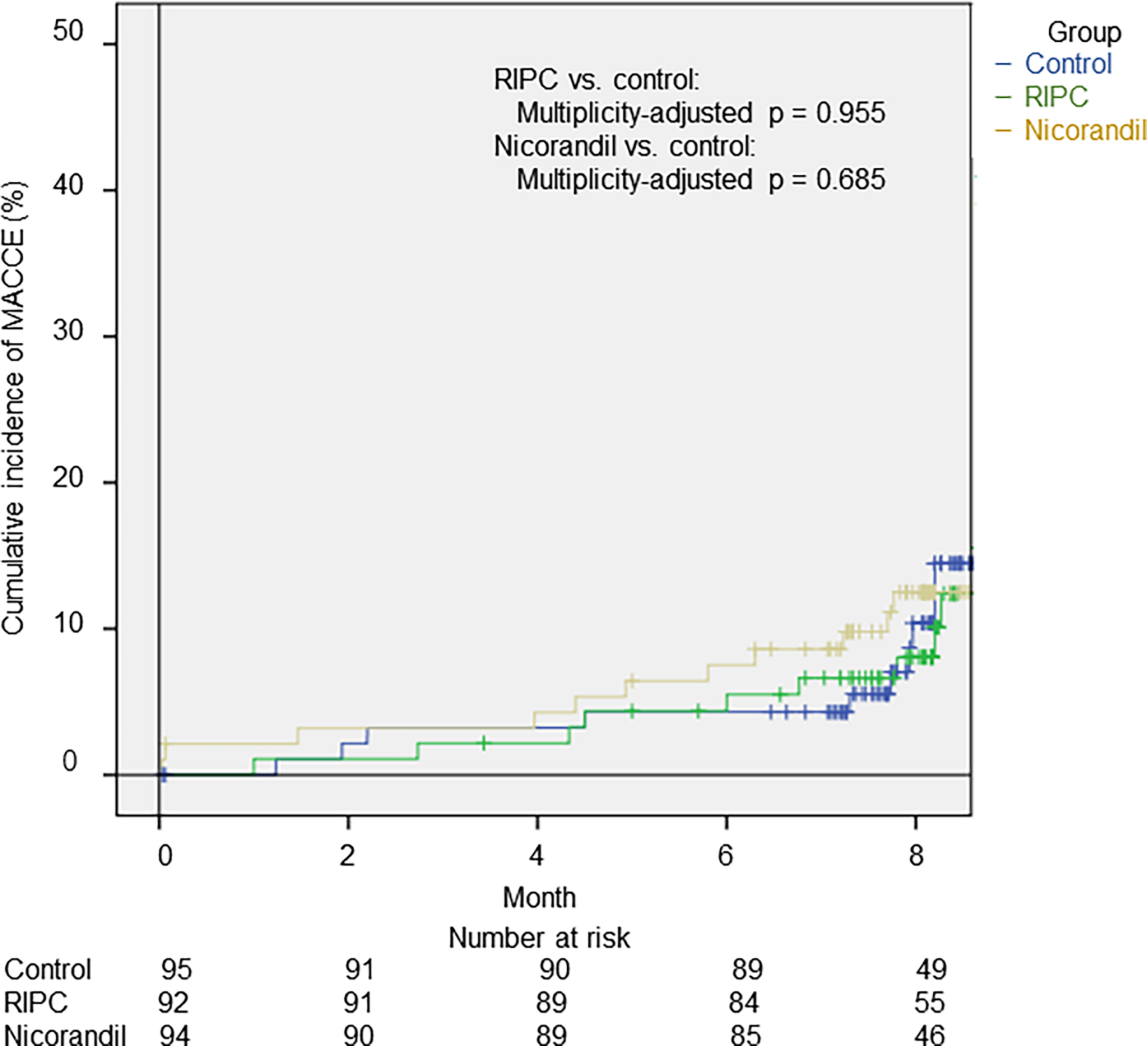
Incidence of MACCE in patients aged > 65 years. MACCE included cardiovascular or non-cardiovascular death, admission for acute coronary syndrome, any revascularization, and admission for heart failure. MACCE, major adverse cardiac or cerebrovascular events; RIPC, remote ischemic preconditioning.

As shown in Fig 3, Kaplan–Meier estimates by the log-rank test for adverse clinical events at 8 months after PCI were not significantly different between the control and nicorandil groups (11/94 [12.5%]; multiplicity-adjusted *p* = 0.685), or between the control and RIPC groups (8/95 [10.4%] vs. 7/92 [8.1%]; multiplicity-adjusted *p* = 0.955). Furthermore, in Cox regression analysis in the older cohorts, the incidence of adverse clinical events at 8 months after PCI was not significantly different between the control and nicorandil groups (HR: 1.38; 95% CI: 0.56 to 3.57), or between the control and RIPC groups (HR: 0.87; 95% CI: 0.31 to 2.42) (Table 2).

## Discussion

In this post-hoc analysis of a multicenter, randomized controlled trial, we demonstrated that intravenous nicorandil significantly reduced the incidence of pMI following PCI in older patients with stable CAD compared with the control group. However, RIPC failed to show a significant reduction in pMI. Nicorandil or RIPC did not reduce ischemic complications during elective PCI and clinical adverse events within 8 months after PCI, similar to the principal study [24].

This sub-analysis showed a significant effect of intravenous nicorandil on pMI in older patients with stable CAD. However, in sub-analysis of patients aged ≤65 years, the incidence of pMI was not significantly different between the nicorandil and control groups. In patients with advanced age, microvascular function is considered to be impaired [31]. Nicorandil is a unique hybrid pharmacological agent of an adenosine triphosphate-sensitive potassium channel opener and nicotinamide nitrate. Therefore, an explanation for the beneficial effect of nicorandil is that nicorandil might compensate for a decline in cardiac mitochondrial function and ability of nitric oxide production in older patients. Notably, the route of nicorandil administration was different among previous studies. Single-center studies showed that intravenous nicorandil reduced pMI or the slow flow phenomenon following PCI in patients who had elective and emergent PCI [15,16,32,33]. However, the effect of intracoronary nicorandil is controversial [17,18,19]. In our study, intravenous nicorandil administration before and after PCI might have increased tissue concentrations of nicorandil before PCI, and this could have contributed to the protective effect against pMI.

This sub-analysis also showed that pre-procedural upper limb RIPC moderately, but not significantly, reduced pMI in older patients with stable CAD. There are several possible reasons for this lack of significance. One reason is the small sample size of this sub-analysis. This sub-analysis might not have been sufficiently powered for differences that we expected, similar to our previous study. Another reason for this lack of significance could be an influence of age on the effect of RIPC. A meta-analysis showed that age was negatively correlated with a reduction in acute kidney injury by RIPC [34]. Therefore, various comorbidities and/or confounders could influence the effect of RIPC on target organs. However, further study to identify the populations who benefit from RIPC is warranted because RIPC is easily delivered, safe, and has a low cost.

The present study also showed that the overall incidence of pMI was 43.3% as determined with cardiac troponin T and 30.1% as determined with CK-MB. In line with our results, previous studies have shown that the incidence of pMI following elective PCI determined with cardiac troponin is higher than that determined with CK-MB when using the cut-off established by the Third Universal Definition of Myocardial Infarction [35]. For example, Li et al. [36] reported that the incidence of elevated biomarkers after elective PCI in patients with stable angina pectoris using the defined cut-off (>5× the upper reference limit) was 32.9% using cardiac troponin T and 14.5% using CK-MB. The present study demonstrated that the incidence of pMI in the control group was 53.7% based on the Third Universal Definition of Myocardial Infarction [35], which was relatively higher that that in other studies (15%–63%) [13,25,26,29,30,37,38,39,40,41]. Several factors in our study may explain the high incidence of pMI. First, the present study included patients aged >65 years. Older age is an important patient-related risk factor for pMI [5]. Second, the higher prevalence of diabetes mellitus (53.5%) in our study than previous studies may have been associated with the higher presence of lipid-rich plaques at target lesions. This could lead to distal embolization of atheromatous material or occlusion of small side branches despite angiographic success. In fact, the highest reported incidence of pMI was 63% in patients with diabetes mellitus [41]. This difference in risk factors in the populations of different studies could have resulted in the greater incidence of pMI in our study.

This study has several limitations. First, this study was a post-hoc analysis of a randomized, controlled trial. Although the RINC study was a large randomized, controlled trial of RIPC in patients with elective PCI, the study sample size was relatively small because of subgroup analysis. A small sample size might have resulted in low statistical power in analysis of intravenous RIPC in this study. Second, this sub-analysis attempted to compare the nicorandil and RIPC groups separately with the control group. However, two treatment comparisons (i.e., nicorandil vs. control and RIPC vs. control) were not independent because of the same control group. Therefore, we should not consider the study results as separate independent comparisons of the two forms of treatment (nicorandil and RIPC). Third, data regarding side branch occlusion, which is one of the causes of pMI during PCI, were not available in the current study. Although final TIMI grades did not differ among treatment groups, side branch occlusion may have affected the result of this sub-analysis.

## Conclusions

In conclusion, in this secondary analysis of the RINC study, periprocedural intravenous nicorandil significantly reduced the incidence of pMI in older patients with stable CAD. Our study suggests that intravenous nicorandil is effective in myocardial protection for stable PCI in these patients. Further investigation using a multicenter, prospective study is required to evaluate the effect of nicorandil on pMI following PCI in older patients.

## Supporting information

S1 CONSORT Checklist (DOC)

S1 Protocol (DOC)

S1 Data Set Full data set. (XLSX)

S1 Table Baseline characteristics of patients aged ≤65 years. (DOCX)

S1 Fig Incidence of periprocedural myocardial injury in patients aged ≤65 years. (TIF)
