# Supplementary material for: Protective effect of nicorandil on myocardial injury following percutaneous coronary intervention in older patients with stable coronary artery disease: Secondary analysis of a randomized, controlled trial (RINC)
Source: PLoS One. 2018 Apr 16;13(4):e0194623. doi: 10.1371/journal.pone.0194623 (PMC5901776; doi:10.1371/journal.pone.0194623)
Supplement: S1 Protocol — (DOC) [file pone.0194623.s002.doc]

Trial Protocol

Cardiac Preconditioning Effect of Remote Ischemia and Nicorandil in Patients Undergoing Elective Percutaneous Coronary Intervention.

Brier title: RINC study

Date of disclosure of the study information

June 1^st^, 2011

Funding source: This study is funded by the Okayama Medical Foundation, which is a nonprofit institution. It is not involved in the design of the protocol, the conduct of the study, or the analyses or reporting of the data.

A person in charge:

Hiroshi Ito

Department of Cardiovascular Medicine

Okayama University Graduate School of Medicine, Dentistry and Pharmaceutical Sciences

Address: 2-5-1, Shikata-cho, Kita-ku, Okayama

TEL: (+81) 86-235-7349

[itomd@md.okayama-u.ac.jp](mailto:itomd@md.okayama-u.ac.jp)

Contents

I. Summary of study plan

II. Background of study plan

III. Study plan

1. Purpose

2. Study population

3. Consent

4. Interventions

5. Methods

6. Endpoints

7. Cancellation and withdrawal

8. Study period

9. Statistics

10. Data management and analysis

11. Ethical consideration

12. Methods of plan change

13. A person in charge and office

14. Funding sauce

15. Collaboration with other scientists or research institutions

I. Summary of study plan

To investigate efficacy and safety of remote ischemic preconditioning (RIPC) and intravenous nicorandil administration before percutaneous coronary intervention (PCI) procedure for patients with stable myocardial ischemia by multicentre randomized controlled trial.

II. Background of study plan

The effect of RIPC and nicorandil on periprocedural myocardial damage (pMD) in patients with planned PCI remains controversial. The aim of this randomized trial was to evaluate the effect of RIPC or nicorandil on pMD following PCI in patients with stable coronary artery disease (CAD) compared with a control group.

III. Study plan

1. Purpose

The aim of this study was to investigate the efficacy of RIPC or intravenous nicorandil on pMD in patients with stable CAD undergoing elective PCI by a randomized, multicentre trial. To improve the reproducibility of RIPC, we have developed a device, which can automatically repeat ischemia and reperfusion in the upper limbs.

2. Study population

Eligible patients were adults (>20 years old) who were diagnosed with stable CAD including silent myocardial ischemia and stable angina and planned to have elective PCI. All patients underwent coronary angiography before enrolment of this study. Indication of PCI was evaluated according to the guideline for elective percutaneous coronary intervention in patients with stable coronary artery disease from Japanese society of cardiology.

Exclusion criteria

1) Acute coronary syndrome patient undergoing emergency PCI.

2) Patient corresponds to contraindication of intravenous nicorandil administration.

3) Patient undergoing PCI to chronic total occlusion lesion or patients undergoing PCI with rotablator.

4) Diabetes patient treated with glibenclamide.

5) Patient with AV shunts on the arms.

6) Patient whose prognosis is regarded as less than 12 months.

7) Patient considered being improper to this study by attending doctor.

3. Consent

1) Written informed consent is needed to be received.

2) If patients do not have ability of judgment, informed consent cannot be received.

Informed Consent Form

1 Purpose

2 Efficacy and side effect

3 Alternative treatment options

4 NO disadvantage by rejection

5 Withdrawal rights

6 Ethics

4. Interventions

1) Control: Subjects who do not undergo any pre-treatment before PCI.

2) RIPC: Subjects who undergo RIPC pre-treatment induced by three 5-minute inflations of a blood pressure cuff to 200mmHg around the upper arm, followed by 5-minute intervals of reperfusion 1 hour before PCI.

3) Nicorandil: Subjects who undergo intravenous 4mg nicorandil administration as a bolus 1 hour before PCI, followed by 6mg/hour.

5. Methods

Patients are randomly assigned in a 1:1:1 ratio to control, intravenous nicorandil (6 mg/h), or RIPC. Randomization is conducted by the Clinical Trials Unit based at Okayama University via a secure website and is stratified by the centre using random permuted blocks. In the patients who are assigned to the RIPC group, 5-minute inflation of a blood pressure cuff to 200 mmHg around the upper arm, followed by 5-minute deflation of a cuff to 0 mmHg is performed three times at least 1 hour before PCI.

This procedure is automatically performed by a newly developed automated continuous blood pressure device (FB-270; Fukuda Denshi, Tokyo, Japan). In the patients who are assigned to the nicorandil group, 4 mg of nicorandil is intravenously administered for 5 minutes at least 1 hour before PCI, followed by continuous infusion of nicorandil (6 mg/h) for at least 8 hours. Stopping the infusion is dependent on the practice of each hospital. Patients in the control group do not receive any additional pretreatment before PCI.

Blood samples for high-sensitive cardiac troponin T (cTnT) and creatine kinase myocardial band (CK-MB) are collected at 12 and 24 hours after PCI. To avoid inter-hospital variation of high-sensitive cTnT and CK-MB levels, these markers are evaluated at a single institution (SRL Inc. Hachioji Laboratory, Tokyo, Japan). Study investigators who collect and analyse the data are blinded to the treatment assignments.

6. End points

The primary end point is the incidence of pMD following PCI. The definition of pMD is as follows: an elevation in high sensitive cTnT levels >0.07 ng/ml (5 × 99th percentile upper reference limit); or CK-MB levels >10 ng/ml and CK-MB/creatinine kinase levels >5%, at 12 hours or 24 hours after PCI. In case of patients being discharged before regular assessment, these cardiac biomarkers were assessed at discharge.

At the time of planning the protocol, myocardial injury was defined as an elevation in levels of non-high sensitive cTnT >0.03 ng/ml; or CK-MB levels >10 ng/ml and CK-MB/creatinine kinase levels >5%, at 12 or 24 hours after PCI. However, at the time of starting this study, cTnT which measured in core laboratory was changed from non-high sensitive cTnT to high sensitive cTnT. Therefore, cut off value of cTnT was revised based on the diagnostic criteria for myocardial infarction with PCI from the third universal definition of myocardial infarction, which had changed in 2012.

The secondary end points are ischemic events during PCI, including the procedural success rate, chest pain during PCI, ST segment change on an electrocardiogram (>1 mV) during PCI, ventricular arrhythmia needed for cardioversion during PCI, and final TIMI grade. We also study adverse clinical events at 8 months after PCI as a secondary end point. Adverse clinical events included cardiovascular or non-cardiovascular death, admission for acute coronary syndrome, any revascularization, and admission for heart failure.

7. Cancellation and withdrawal

1) Declaration of withdraw

2) Adverse events

3) Requirements of emergency treatment

4) Complication of PCI procedure

5) Termination of clinical trial

6) Not eligibility after enrolment

7) Judgement of attending doctor

8. Study period

Between 1^st^ June, 2011 and 28^th^ February, 2014

9. Statistics

Efficacy analyses are performed in the full analysis set, which is defined as all randomized patients who received any protocol treatment and PCI, and their eligibility is confirmed. Although the supplemental per protocol set for efficacy analyses is also defined. Safety analyses are conducted for all randomized patients. In efficacy analyses, patients with abnormal cTnT, CK-MB, and/or CK-MB/CK values (high sensitive cTnT: >0.014 ng/ml, CK-MB: >5 ng/ml, and CK-MB/creatinine kinase: >5%) are additionally analysed.

We assumed that the proportion of pMD following PCI was 30% in the control group and 12% in the RIPC and nicorandil groups. Multiplicity adjustment was not applied because the RIPC and nicorandil groups were separately compared and interpreted with the control group. To assure that the two-sided significance level was 5% and the power was 90%, 106 patients in each group were required. Assuming that approximately 20% of patients would drop out and/or abnormal values would be detected at pre-PCI, we set the sample size as 133 patients per group.

In primary analysis, Fisher’s exact test is applied to compare proportions of pMD following PCI between the RIPC and control groups or the nicorandil and control groups. The risk differences of proportions between groups and their 95% confidence intervals (CIs) were calculated. Additionally, a logistic regression model is used to calculate odds ratios (ORs) between study groups with adjustment for age (<65 or >65 years old), sex, and with or without chronic kidney disease (estimated glomerular filtration rate [eGFR] at baseline <60 or >60 ml/min/1.73 m2). The Mann–Whitney U test is applied to compare the levels of high sensitive cTnT following PCI between the RIPC and control groups or nicorandil and control groups.

The same analyses of the primary end point are applied to ischemic events during PCI. For adverse clinical events at 8 months after PCI, the Kaplan–Meier estimate is used by treatment group and compared using the log-rank test. Cox’s proportional hazard model is used to estimate hazard ratios (HRs) between treatment groups. We also evaluate the effect of RIPC and intravenous nicorandil for the primary end point in subgroups defined by the following baseline characteristics: age (<65 years old or >65 years old), sex, with or without chronic kidney disease (eGFR at baseline <60 ml/min/1.73 m2 or eGFR at baseline >60 ml/min/1.73 m2), with or without diabetes, smoking history (current smoker, ex-smoker, or never smoker) and AHA-ACC classification of PCI lesion (types A and B1 or B2 and C). We also use a repeated-measures linear mixed-effects model to assess troponin and CK-MB as continuous variables. To account for non-normality, these endpoints are natural log transformed and use as dependent variables. Independent variables in this model are log transformed baseline troponin or CK-MB, age category, sex, chronic kidney disease status, treatment arm, scheduled visit as a class variable (12h, 24h) and the interaction between the arm and the visit, with the use of an unstructured covariance matrix. We use residual maximum likelihood (REML) method for estimation. The Kenward–Roger approximation is used to estimate denominator degrees of freedom. Least square mean estimates and their 95% confidence intervals are exponentially back-transformed.

Continuous variables are presented as means ± standard deviation or as medians with interquartile range. Categorical variables are presented as numbers and ratios (%). Continuous variables are compared with the use of analysis of variance or the Kruskal–Wallis test for nonnormally distributed data. All analyses are performed with SAS version 9.3 (SAS Institute Inc., Cary, NC, USA). A p value of less than 0.05 was considered statistically significant.

10. Data management and analysis

EPS Associates Co., Ltd.

2-23 Shimomiyabicho, Shinjuku-ku, Tokyo, 162-0822, Japan

TEL : +81-3-3868-7200 / FAX : +81-3-3868-7201

E-mail: info@eps-associates.com

11. Ethical consideration

All participants provide written informed consent before enrolling.

This study is conducted according to the principles expressed in the Declaration of Helsinki.

This study was approved by Okayama University Graduate School of Medicine, Density and Pharmaceutical Sciences and Okayama University Hospital, Ethics Committee, and Ethics Committees in each research facility.

The study is registered at the UMIN Clinical Trials Registry, June 2011 (UMIN000005607), https://upload.umin.ac.jp/cgi-open-bin/ctr_e/ctr_view.cgi?recptno=R000006626

12. Methods of plan change

When needed, investigators are going to make a discussion and decision of plan change.

13. A person in charge and office

Hiroshi Ito

Department of Cardiovascular Medicine

Okayama University Graduate School of Medicine, Dentistry and Pharmaceutical Sciences

Address: 2-5-1, Shikata-cho, Kita-ku, Okayama

TEL: (+81) 86-235-7349

[itomd@md.okayama-u.ac.jp](mailto:itomd@md.okayama-u.ac.jp)

14. Funding sauce

This study is funded by the Okayama Medical Foundation, which is a nonprofit institution. It is not involved in the design of the protocol, the conduct of the study, or the analyses or reporting of the data.

15. Collaboration with other scientists or research institutions

1) Measurement institutions

SRL Hachioji laboratory

Hachioji Komiya 51, Tokyo 192-8535

2) Research facilities

RINC collaborators:

Kentaro Ejiri, MD; Toru Miyoshi, MD, PhD; Kunihisa Kohno, MD, PhD; Makoto Nakahama, MD, PhD Masayuki Doi, MD, PhD; Mitsuru Munemasa, MD, PhD; Masaaki Murakami, MD, PhD; Atsushi Takaishi MD, PhD; Yusuke Kawai, MD, PhD; Tetsuya Satoh, MD, PhD; Katsumasa Satoh, MD, PhD; Takefumi Oka, MD, PhD; Natsuki Takahashi, MD, PhD; Satoru Sakuragi, MD, PhD; Atsushi Mima, MD, PhD; Kenki Enko, MD, PhD; Shingo Hosogi, MD, PhD; Seiji Nanba, MD, PhD; Ryoichi Hirami, MD, PhD, Yasukazu Fujiwara, MD; Yoshimasa Morimoto, MD; Shunji Suemaru, MD; and Toshiaki Yamanaka, MD; Kazufumi Nakamura, MD, PhD; Hiroshi Ito, MD, PhD

Affiliations:

Department of Cardiovascular Medicine, Okayama University Graduate School of Medicine, Density and Pharmaceutical Sciences, Okayama, Japan

K. Ejiri, T. Miyoshi, K. Kohno, K. Nakamura, H. Ito

Department of Cardiology, Fukuyama City Hospital, Hiroshima, Japan

M. Nakahama

Department of Cardiology, Kagawa Prefectural Central Hospital, Kagawa, Japan

M. Doi

Department of Cardiology, Okayama Medical Centre, Okayama, Japan

M. Munemasa

Department of Cardiology, Okayama Heart Clinic, Okayama, Japan

M. Murakami

Department of Cardiology, Mitoyo General Hospital, Kagawa, Japan

A. Takaishi

Department of Cardiology, Ehime Prefectural Central Hospital, Ehime, Japan

Y. Kawai, T. Yamanaka

Department of Cardiology, Okayama City General Medical Centre, Okayama, Japan

Y. Kawai

Department of Cardiology, Okayama Red Cross Hospital, Okayama, Japan

T. Satoh

Department of Cardiology, Fukuyama Cardiovascular Hospital, Hiroshima, Japan

K. Satoh, Y. Morimoto

Department of Cardiology, Tsuyama Central Hospital, Okayama, Japan

T. Oka, T. Yamanaka

Department of Cardiology, Matsuyama Shimin Hospital, Ehime, Japan

T. Natsuki

Department of Cardiology, Iwakuni Clinical Centre, Yamaguchi, Japan

S. Sakuragi

Department of Cardiology, Saiseikai Imabari Hospital, Ehime, Japan

A. Mima

Department of Cardiology, Onomichi Municipal Hospital, Hiroshima, Japan

K. Enko

Department of Cardiology, Kohchi Health Sciences Centre, Kohchi, Japan

S. Hosogi

Department of Cardiology, Okayama Rosai Hospital, Okayama, Japan

S. Nanba

Department of Cardiology, Himeji Red Cross Hospital, Hyogo, Japan

R. Hirami

Department of Cardiology, Kagawa Rosai Hospital, Kagawa, Japan

Y. Fujiwara
