# Supplementary material for: Protective effect of nicorandil on myocardial injury following percutaneous coronary intervention in older patients with stable coronary artery disease: Secondary analysis of a randomized, controlled trial (RINC)
Source: PLoS One. 2018 Apr 16;13(4):e0194623. doi: 10.1371/journal.pone.0194623 (PMC5901776; doi:10.1371/journal.pone.0194623)
Supplement: S1 Table — (DOCX) [file pone.0194623.s004.docx]

**S2 Table. Baseline characteristics in patients aged ≦ 65 years.**

|  | Control group  (n=38) | RIPC group (n=36) | Nicorandil group(n=35) | p value |
| --- | --- | --- | --- | --- |
| Age-yr. | 57.6(7.8) | 58.4(6.7) | 58.5(5.7) | 0.83 |
| Male-n (%) | 33(86.8) | 33(91.7) | 30(85.7) | 0.52 |
| Body mass index (kg/m^2^) | 24.2(3.6) | 24.8(3.2) | 25.8(3.5) | 0.15 |
| Angina symptom-n (%) |  |  |  |  |
| symptomatic | 29(76.3) | 28(77.8) | 23(65.7) | 0.45 |
| asymptomatic | 9(23.7) | 8(22.2) | 12(34.3) |  |
| Prior diagnoses-n (%) |  |  |  |  |
| Diabetes Mellitus | 17(44.7) | 16(44.4) | 14(40.0) | 0.9 |
| Hypertension | 28(73.7) | 24(66.7) | 29(82.9) | 0.29 |
| Dyslipidemia | 31(81.6) | 32(88.9) | 30(85.7) | 0.67 |
| CCV event history-n (%) | 19(50.0) | 9(25.0) | 16(45.7) | 0.07 |
| Smoking history-n (%) |  |  |  |  |
| Current smoker | 11(28.9) | 7(19.4) | 6(17.1) | 0.46 |
| Ex-smoker | 16(42.1) | 21(58.3) | 22(62.9) |  |
| Echocardiographic parameters at randomization |  |  |  |  |
| LVEF (%) | 63.9(8.7) | 63.7(10.8) | 62.2(9.4) | 0.73 |
| E/e | 11.2(3.7) | 11.6(4.3) | 10.9(3.5) | 0.79 |
| Laboratory data at randomization |  |  |  |  |
| Hemoglobin (g/dl) | 13.6(1.8) | 14.3(1.6) | 14.0(1.6) | 0.24 |
| Platelet count (10^4^/µl) | 21.8(6.4) | 21.0(5.6) | 20.8(4.7) | 0.75 |
| Total cholesterol (mg/dl) | 162.0[136.5-199.0] | 168.5[147.5-204.3] | 163.0[138.5-184.5] | 0.66 |
| Serum creatinine (mg/dl) | 0.81[0.69-0.99] | 0.80[0.73-0.90] | 0.79[0.69-1.0] | 0.98 |
| eGFR (ml/min/1.73 cm^2^) | 72.9(16.0) | 75.0(15.9) | 73.4(18.8) | 0.85 |
| Hemoglobin A1C (%) | 5.6[5.2-6.3] | 5.8[5.3-7.2] | 5.7[5.2-6.7] | 0.38 |
| C-reactive protein (mg/dl) | 0.10[0.03-0.20] | 0.09[0.04-0.19] | 0.10[0.04-0.19] | 0.97 |
| Brain natriuretic peptide (pg/ml) | 21.4[6.9-54.4] | 24.7[11.8-59.2] | 27.5[12.3-44.5] | 0.94 |
| Myocardial biomarker at randomization |  |  |  |  |
| Cardiac troponin T (ng/ml) | 0.010[0.007-0.018] | 0.008[0.007-0.014] | 0.013[0.006-0.029] | 0.68 |
| CK-MB (ng/ml) | 3.4[2.2-4.9] | 3.4[2.5-3.8] | 3.0[2.0-5.0] | 0.84 |
| Medications at randomization-no. (%) |  |  |  |  |
| Antiplatelets | 38(100) | 35(97.2) | 34(97.1) | 0.32 |
| β-blockers | 19(50.0) | 18(50.0) | 20(57.1) | 0.78 |
| ACEIs/ARBs | 23(60.5) | 18(50.0) | 24(68.6) | 0.28 |
| Calcium channel blockers | 13(34.2) | 10(27.8) | 16(45.7) | 0.28 |
| Statins | 33(86.8) | 31(86.1) | 29(82.9) | 0.88 |
| Procedure characteristics |  |  |  |  |
| Target vessel-no. (%) |  |  |  |  |
| LAD | 13(34.2) | 17(47.2) | 13(37.1) | 0.49 |
| LCX | 8(21.1) | 8(22.2) | 5(14.3) | 0.66 |
| RCA | 16(42.1) | 10(27.8) | 14(40.0) | 0.39 |
| multiple | 1(2.6) | 1(2.8) | 3(8.6) | 0.39 |
| AHA-ACC classification-no. (%) |  |  |  |  |
| Type A | 10(26.3) | 5(13.9) | 6(17.1) | 0.79 |
| Type B1 | 10(26.3) | 13(36.1) | 9(25.7) |  |
| Type B2 | 14(36.8) | 13(36.1) | 16(45.7) |  |
| Type C | 4(10.5) | 5(13.9) | 4(11.4) |  |
| Amount of contrast medium (ml) | 102.0  (50.8) | 113.6  (48.1) | 111.3  (40.8) | 0.57 |
| Puncture site, n/total n (%) |  |  |  |  |
| Radial artery | 23/37  (62.2)* | 21/36  (58.3) | 20/32  (62.5)** | 0.28 |
| Brachial artery | 7/37(18.9)* | 2/36(5.6) | 3/32(9.4)** |  |
| Femoral artery | 7/37(18.9)* | 13/36(36.1) | 9/32(28.1)** |  |
| Catheter size, n/total n (%) |  |  |  |  |
| 6 Fr | 31/37(83.8)* | 29/36(80.6) | 28/32(87.5)** | 0.63 |
| 7 Fr | 5/37(13.5)* | 7/36(19.4) | 4/32(12.5)** |  |
| 8 Fr | 1/37(2.7)* | 0 | 0 |  |
| Details of device |  |  |  |  |
| No. of stents used | 1[1-2] | 1[1-2] | 1[1] | 0.72 |
| Drug-eluting stent, n/total n (%) | 38/41(92.7) | 40/47(85.1) | 32/39(82.1) | 0.35 |
| Stent diameter (mm) | 3[2.5-3.5] | 3[2.5-3.5] | 3[2.5-3.5] | 0.86 |
| Stent length (mm) | 18[15-24] | 18[14-28] | 18[15-24] | 0.82 |
| Stent inflation time (second) | 15[9-20] | 15[10-20] | 15[10-20] | 0.7 |
| Post dilation (%) | 18/38(47.4) | 29/36(80.6) | 25/35(71.4) | 0.008 |
| Post dilation time (second) | 15[10-34] | 15[10-30] | 15[10-30] | 0.77 |
| Maximum dilatation pressure (atm) | 16.1(4.0) | 15.4(4.4) | 16.5(4.0) | 0.60 |

Data are mean (standard deviation), n (%), or median [interquartile range]. RIPC, remote ischemic preconditioning; CCV, cardio-cerebrovascular; LVEF, left ventricular ejection fraction; E, peak velocity of early diastolic filling wave; e′, mitral annulus velocity; eGFR, estimated glomerular filtration rate; CK, creatine kinase; ACEI, angiotensin-converting enzyme inhibitor; ARB, angiotensin II receptor blocker; LAD, left anterior descending artery; LCX, left circumflex artery; RCA, right coronary artery; AHA, American Heart Association; ACC, American College of Cardiology; Fr, French; atm, atmospheres.

*one patient datum was uncollected. **three patient data were uncollected.
