# Supplementary figures and images for: Protective effect of nicorandil on myocardial injury following percutaneous coronary intervention in older patients with stable coronary artery disease: Secondary analysis of a randomized, controlled trial (RINC)
Source: PLoS One. 2018 Apr 16;13(4):e0194623. doi: 10.1371/journal.pone.0194623 (PMC5901776; doi:10.1371/journal.pone.0194623)

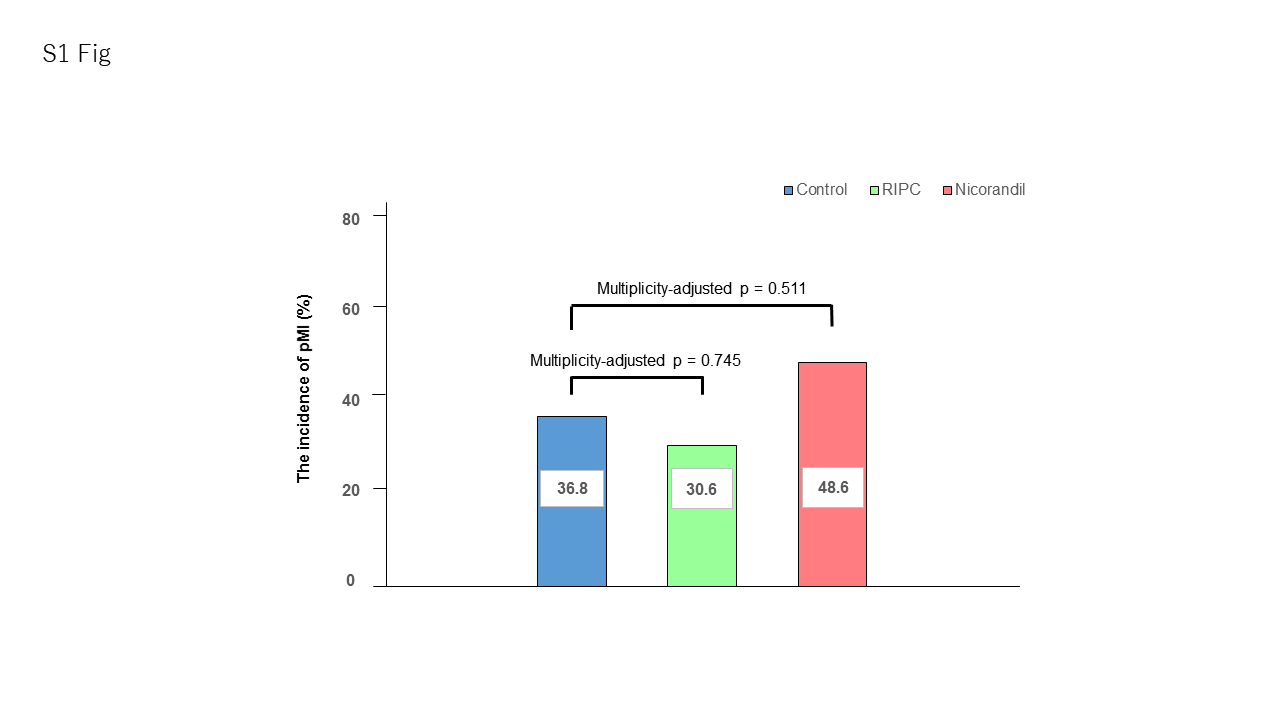

Supplement: S1 Fig — (TIF) [file pone.0194623.s005.TIF]
